# Supplementary material for: Investigation of the Key Genes Associated with Anthocyanin Accumulation during Inner Leaf Reddening in Ornamental Kale (Brassica oleracea L. var. acephala)
Source: Int J Mol Sci. 2023 Feb 2;24(3):2837. doi: 10.3390/ijms24032837 (PMC9917897; doi:10.3390/ijms24032837)
Supplement: Supplementary file 1 [file ijms-24-02837-s001.zip › ijms-2188102-supplementary.pdf]

Table S1. Summary of transcriptome sequencing data.

Sample	Raw Data Read	Raw Data Base	Valid Data Read	Valid Data Base	Valid Ratio (reads)	Q20%	Q30%	GC content%
S0_1	45,944,336	6.89G	45,381,766	6.81G	98.78	99.68	96.89	46.5
S0_2	46,147,772	6.92G	45,672,436	6.85G	98.97	99.63	96.79	46.5
S0_3	56,896,248	8.53G	56,095,986	8.41G	98.59	99.5	95.34	46.5
S1_1	46,366,224	6.95G	44,788,004	6.72G	96.6	99.36	95.82	46.5
S1_2	46,445,136	6.97G	45,910,946	6.89G	98.85	99.52	96.45	47
S1_3	52,275,366	7.84G	49,349,172	7.40G	94.4	99.38	96.07	46.5
S2_1	49,100,766	7.37G	48,519,316	7.28G	98.82	99.54	96.43	46.5
S2_2	41,965,700	6.29G	41,491,120	6.22G	98.87	99.48	96.44	47
S2_3	48,852,286	7.33G	48,337,902	7.25G	98.95	99.73	95.36	47

Table S2. Statistics date of the alignment between valid reads and reference genome.

Sample	Valid reads	Mapped reads	Unique Mapped reads	Multi Mapped reads	PE Mapped reads	Reads map to sense strand	Reads map to antisense strand	Non-splice reads	Splice reads
S0_1	45381766	40305253(88.81%)	29492498(64.99%)	10812755(23.83%)	36481492(80.39%)	19368150(42.68%)	19415396(42.78%)	22195630(48.91%)	16587916(36.55%)
S0_2	45672436	40507092(88.69%)	29725357(65.08%)	10781735(23.61%)	36351772(79.59%)	19439062(42.56%)	19506384(42.71%)	21722037(47.56%)	17223409(37.71%)
S0_3	56095986	49495423(88.23%)	35691317(63.63%)	13804106(24.61%)	44167792(78.74%)	23744425(42.33%)	23816119(42.46%)	27167921(48.43%)	20392623(36.35%)
S1_1	44788004	39018973(87.12%)	28689454(64.06%)	10329519(23.06%)	33163544(74.05%)	18732057(41.82%)	18778870(41.93%)	20953209(46.78%)	16557718(36.97%)
S1_2	45910946	40504701(88.22%)	28875734(62.90%)	11628967(25.33%)	36193334(78.83%)	19192335(41.80%)	19254088(41.94%)	21822790(47.53%)	16623633(36.21%)
S1_3	49349172	42871978(86.87%)	31706010(64.25%)	11165968(22.63%)	35499908(71.94%)	20556663(41.66%)	20617923(41.78%)	23911105(48.45%)	17263481(34.98%)
S2_1	48519316	42940895(88.50%)	31197250(64.30%)	11743645(24.20%)	38401442(79.15%)	20546864(42.35%)	20603463(42.46%)	23729446(48.91%)	17420881(35.91%)

S2_2	41491120	36582367(88.17%)	26359475(63.53%)	10222892(24.64%)	32455674(78.22%)	17435004(42.02%)	17477657(42.12%)	20059626(48.35%)	14853035(35.80%)
S2_3	48337902	42983832(88.92%)	27778574(57.47%)	15205258(31.46%)	39138142(80.97%)	19485352(40.31%)	19566866(40.48%)	24203847(50.07%)	14848371(30.72%)

Table S3. Reference genes and primers used for quantitative real-time PCR analysis.

Locus	Primer sequence (5'–3')
4CL3 (*Bo6g099190*)	F: GAGATGAAGTGGCTGGAGAAG<br>R: TGGGAATGGAGGGAACAAAG
C4H (*Bo5g052100*)	F: GTATGTGCCGTTTGGTGTTG<br>R: GTATCCACTTTAGACTGTCCCG
CHI (*Bo9g177250*)	F: ATCACTTACCGTTTCTCAGCC<br>R: TCCCTACCACATTCGCATTC
DFR (*Bo2g116380*)	F: TCGGGATTCATCGGTTCATG<br>R: TTCGTCAGATAAATCGGTGGG
DFR (*Bo9g058630*)	F: AGCCGATTTATCTGACGAAGG<br>R: TTATCCCCAACACTCCATTCAC
DXS (*Bo7g103850*)	F: CGCATGTGGTTCAGTTTCTTG<br>R: GCTATATGAGACGGCATAAGTCC
F3′H (*Bo9g174880*)	F: GCCCGCACTTGATTGTTTAG<br>R: CATGTCCGTGTGCTTTTGATC
F3H (*Bo8g081770*)	F: ACCGTGTATCCGCTTAAAGTG<br>R: TCCTTGTGGTCATGCTCTTC
TT19 (*Bo9g161480*)	F: GCTGATGTTGAGGTGGGTTAC<br>R: TTGTCGTATATGTCCAAGACCAC
Actin (*Bo1g116200*)	F: GGTCGTGACCTTACTGATTACCTCA<br>R: GAAGTCTCCATCTCCTGCTCGT

Table S4. Positive related DEGs in the gene co-expression analysis.

Data1	Data2	rho	p-value	Data1	Data2	rho	p-value
Bo8g081770	Bo9g177250	0.9924252	1.23631E-07	4CL3	MSTRG.20312	0.8709524	0.002235517
Bo3g023240	Bo8g081770	0.986839	8.50121E-07	4CL3	Bo9g017560	0.8694323	0.002325439
Bo8g081770	MSTRG.22323	0.9797221	3.83286E-06	4CL3	Bo3g065330	0.8677186	0.002429778
Bo3g023240	Bo9g177250	0.978684	4.56008E-06	4CL3	ATDFD	0.8671983	0.002462081
4CL3	MSTRG.11229	0.9765148	6.3879E-06	Bo5g030290	Bo8g081770	0.8638904	0.002674444
Bo2g055220	Bo9g177250	0.9758722	7.01644E-06	4CL3	Bo2g057490;Bo2g057500	0.8632824	0.002714802
APX4	Bo9g177250	0.9684753	1.77594E-05	Bo6g121430	Bo8g081770	0.8599344	0.002944597
APX4	Bo8g081770	0.9676253	1.94764E-05	Bo8g077260	Bo8g081770	0.8595603	0.00297108
4CL3	Bo8g081770	0.9664892	2.19507E-05	Bo9g177250	GLU1	0.8593887	0.002983284
Bo2g055220	Bo8g081770	0.9644416	2.69604E-05	Bo8g081770	SBPASE	0.8585852	0.003040871
4CL3	MSTRG.22323	0.9642225	2.75403E-05	Bo5g030290	Bo9g177250	0.8579847	0.003084407
Bo9g177250	MSTRG.22323	0.9617281	3.47814E-05	Bo8g081770	Bo9g017560	0.8570926	0.003149882
Bo5g049100	Bo8g081770	0.9613751	3.59048E-05	Bo7g106470	Bo9g177250	0.856419	0.003199944
4CL3	Bo3g091610	0.9613033	3.61362E-05	Bo1g133310	Bo9g177250	0.8508362	0.003636118

4CL3	Bo3g135880	0.9590898	4.38072E-05	Bo1g002260	Bo8g081770	0.8475102	0.003914478
4CL3	Bo7g003670	0.9574443	0.000050209	4CL3	Bo1g133310	0.8455009	0.004089545
Bo5g049100	Bo9g177250	0.949493	9.07293E-05	Bo8g081770	NDF4	0.8439774	0.004225808
4CL3	Bo9g177250	0.9486776	9.58828E-05	Bo8g081770	Bo9g014470	0.8394624	0.004647845
Bo8g081770	CES101	0.9482874	9.84204E-05	Bo9g177250	SBPASE	0.8364782	0.004942085
4CL3	Bo3g023240	0.9479657	0.000100548	4CL3	Bo8g077260	0.8360073	0.004989655
Bo9g177250	CES101	0.9475742	0.000103181	Bo6g121430	Bo9g177250	0.8325175	0.005351946
Bo3g091610	Bo8g081770	0.947267	0.000105281	Bo8g081770	Bo8g082800	0.8317757	0.005431196
Bo1g019620	Bo8g081770	0.9463161	0.00011197	Bo3g149690	Bo8g081770	0.8313386	0.005478279
4CL3	Bo9g018200	0.9439929	0.000129568	4CL3	Bo5g130300	0.8254321	0.006141975
Bo3g065330	Bo8g081770	0.9417442	0.000148377	Bo8g077260	Bo9g177250	0.8248445	0.006210856
Bo8g081770	MSTRG.36038	0.9415423	0.000150155	4CL3	Bo5g009730	0.8234114	0.006381053
Bo8g081770	MSTRG.11229	0.9410402	0.00015464	Bo2g009460	Bo8g081770	0.822205	0.006526753
Bo8g081770	Bo9g018200	0.9396596	0.000167459	4CL3	Bo6g121430	0.8219448	0.006558474
Bo2g057490; Bo2g057500	Bo9g177250	0.9394099	0.000169855	4CL3	Bo8g020530	0.8213475	0.006631689
Bo2g057490; Bo2g057500	Bo8g081770	0.9373892	0.00019014	Bo1g002260	Bo9g177250	0.8201953	0.006774487
Bo3g065330	Bo9g177250	0.9368326	0.000196014	Bo9g177250	NDF4	0.8195352	0.006857235
Bo3g091610	Bo9g177250	0.9339541	0.000228442	Bo8g081770	PEX11D	0.8157112	0.007350128
4CL3	Bo8g082800	0.9336946	0.000231539	Bo9g014470	Bo9g177250	0.8150543	0.00743714
Bo9g177250	MSTRG.36038	0.9310924	0.000264249	Bo3g149690	Bo9g177250	0.8146619	0.007489466
4CL3	Bo2g055220	0.9297283	0.00028263	4CL3	Bo7g106470	0.8145518	0.007504186
Bo1g019620	Bo9g177250	0.9276384	0.000312507	Bo9g017560	Bo9g177250	0.8128625	0.007732563
Bo9g018200	Bo9g177250	0.9268474	0.000324371	4CL3	GLU1	0.8123753	0.007799288
4CL3	APX4	0.9268353	0.000324555	Bo8g082800	Bo9g177250	0.8111671	0.007966459
4CL3	Bo5g049100	0.9258078	0.00034044	Bo8g081770	Bo9g166510;Bo9g166500	0.8107616	0.008023092
Bo3g135880	Bo8g081770	0.925661	0.000342754	Bo3g066140;Bo3g066150	Bo8g081770	0.8010853	0.009457034

4CL3	Bo1g019620	0.9232013	0.000383173	4CL3	Bo1g002260	0.8004936	0.009549945
Bo9g177250	MSTRG.11229	0.917658	0.00048632	Bo5g073010	Bo8g081770	0.7981188	0.009929051
Bo2g064700	Bo8g081770	0.9172802	0.000493987	Bo9g166510;Bo9g166500	Bo9g177250	0.7964083	0.01020832
Bo8g081770	MSTRG.20312	0.9135413	0.000574493	4CL3	SBPASE	0.7950936	0.01042652
ATDFD	Bo8g081770	0.9121911	0.000605692	Bo2g009460	Bo9g177250	0.7932613	0.01073586
Bo3g135880	Bo9g177250	0.9111206	0.000631252	Bo9g177250	PEX11D	0.7931957	0.01074705
4CL3	CES101	0.9101688	0.000654605	4CL3	Bo9g014470	0.7861386	0.01199702
Bo7g003670	Bo8g081770	0.9076008	0.000720615	Bo3g066140;Bo3g066150	Bo9g177250	0.7779657	0.0135625
Bo9g177250	MSTRG.20312	0.9040504	0.00081934	4CL3	NDF4	0.7752907	0.01410315
4CL3	Bo2g064700	0.8963922	0.001063735	4CL3	Bo3g036060	0.7708756	0.01502674
Bo3g036060	Bo9g177250	0.8956779	0.001088838	Bo2g095130	Bo8g081770	0.7697906	0.01525974
Bo2g064700	Bo9g177250	0.8923614	0.001210819	Bo5g073010	Bo9g177250	0.7690496	0.01542023
Bo7g003670	Bo9g177250	0.8912478	0.001253818	Bo8g081440	Bo8g081770	0.7656587	0.01616909
Bo8g020530	Bo9g177250	0.8907101	0.001274958	4CL3	Bo2g009460	0.7656181	0.01617821

4CL3	Bo2g064700	0.8963922	0.001063735	4CL3	Bo5g030290	0.7611993	0.01719031
Bo3g036060	Bo9g177250	0.8956779	0.001088838	Bo8g081440	Bo9g177250	0.7507767	0.01974248
Bo2g064700	Bo9g177250	0.8923614	0.001210819	4CL3	Bo3g149690	0.7490047	0.02019993
Bo7g003670	Bo9g177250	0.8912478	0.001253818	Bo2g095130	Bo9g177250	0.7459851	0.02099552
Bo8g020530	Bo9g177250	0.8907101	0.001274958	Bo4g188460	Bo8g081770	0.7418439	0.02211999
Bo5g009730	Bo8g081770	0.8898346	0.001309903	Bo3g064000	Bo8g081770	0.7356111	0.02388623
Bo3g036060	Bo8g081770	0.8894413	0.001325815	4CL3	PEX11D	0.7330366	0.02464207
Bo5g130300	Bo8g081770	0.8892099	0.001335241	4CL3	Bo5g073010	0.7294424	0.02572336
ATDFD	Bo9g177250	0.8883099	0.001372342	Bo4g188460	Bo9g177250	0.7253444	0.02699371
4CL3	MSTRG.36038	0.8875015	0.001406268	4CL3	Bo3g066140;Bo3g066150	0.7240729	0.02739607
Bo5g009730	Bo9g177250	0.8801997	0.001739569	4CL3	Bo9g166510;Bo9g166500	0.7216064	0.02818778
Bo1g133310	Bo8g081770	0.8795525	0.001771517	Bo3g064000	Bo9g177250	0.7063654	0.03341318
Bo7g106470	Bo8g081770	0.8794198	0.001778117	Bo5g146590	Bo9g177250	0.6753736	0.04589265
Bo8g081770	GLU1	0.8791832	0.001789927	Bo5g146590	Bo8g081770	0.6733114	0.04681498
4CL3	Bo3g064000	0.8780305	0.001848248	4CL3	Bo2g095130	0.6673356	0.04955435

Bo5g130300	Bo9g177250	0.8744428	0.002038179

Table S5. Negative related DEGs in the gene co-expression analysis.

Data1	Data2	rho	p-value	Data1	Data2	rho	p-value
Bo9g177250	MSTRG.15380	-0.9677876	1.91397E-05	Bo5g009840	Bo8g081770	-0.8413437	0.004468643
Bo8g081770	MSTRG.15380	-0.9499618	8.78566E-05	4CL3	Bo3g037630	-0.8401011	0.004586467
Bo9g177250	MSTRG.36902	-0.9290215	0.000292499	4CL3	SKS1	-0.8374456	0.004845347
Bo3g037630	Bo9g177250	-0.9283764	0.000301716	Bo6g032890	Bo9g177250	-0.8364381	0.00494612
Bo6g093040	Bo9g177250	-0.9280117	0.000307015	4CL3	Bo9g153840	-0.833808	0.005215945
Bo9g177250	MSTRG.5945	-0.9278001	0.00031012	4CL3	Bo3g163990	-0.8326499	0.005337883
Bo6g093040	Bo8g081770	-0.9256051	0.000343637	4CL3	RIP2	-0.8301929	0.005602992
Bo9g172240	Bo9g177250	-0.916164	0.000517136	Bo3g004170	Bo8g081770	-0.829579	0.005670599
Bo9g177250	IQD16	-0.9156711	0.000527595	Bo3g184380	Bo9g177250	-0.8287612	0.005761534
Bo4g195410	Bo9g177250	-0.9154704	0.000531896	Bo7g118740	Bo8g081770	-0.8275286	0.005900463
Bo9g177250	ENODL14	-0.9148758	0.00054478	4CL3	Bo4g195410	-0.82746	0.005908262
Bo9g177250	RIP2	-0.912969	0.000587577	4CL3	Bo2g006660	-0.8268015	0.005983478

Bo9g177250	MSTRG.6090	-0.9121967	0.00060556	Bo3g017510	Bo8g081770	-0.8252343	0.0061651
Bo2g006660	Bo9g177250	-0.9111199	0.00063127	4CL3	Bo3g021660	-0.8231152	0.006416612
Bo9g177250	SKS1	-0.9096383	0.00066788	4CL3	IQD16	-0.823024	0.006427598
Bo8g081770	ENODL14	-0.9089557	0.000685235	Bo8g081770	DIN11	-0.8206153	0.006722194
Bo3g163990	Bo9g177250	-0.9035493	0.000833992	Bo1g134740	Bo9g177250	-0.8191428	0.006906741
Bo8g081770	MSTRG.5945	-0.9013876	0.000899309	Bo8g081770	Bo9g181830	-0.8190802	0.006914659
Bo9g153840	Bo9g177250	-0.90053	0.000926178	4CL3	Bo1g039320	-0.8182688	0.007017891
Bo3g037630	Bo8g081770	-0.9003663	0.000931369	4CL3	MSTRG.36902	-0.8177318	0.007086787
Bo8g081770	MSTRG.6090	-0.8981653	0.001003163	4CL3	MSTRG.36727	-0.8141696	0.007555442
4CL3	Bo6g093040	-0.8939807	0.001150137	4CL3	Bo6g072960	-0.8129589	0.007719405
Bo9g177250	MSTRG.36727	-0.8937296	0.001159403	4CL3	Bo6g106910	-0.8085805	0.008332458
Bo9g177250	MSTRG.29785	-0.8934539	0.00116964	Bo7g093130	Bo8g081770	-0.8053634	0.008803364
Bo8g081770	MSTRG.36902	-0.8921127	0.001220331	4CL3	Bo2g006810	-0.8045486	0.00892543
Bo1g039320	Bo9g177250	-0.8918406	0.0012308	Bo8g081770	PFK3	-0.8042555	0.008969613
Bo6g072960	Bo9g177250	-0.89163	0.001238942	Bo8g081770	Bo9g169650	-0.8037834	0.009041099
Bo3g021660	Bo9g177250	-0.8910883	0.001260065	Bo3g153060	Bo9g177250	-0.8031517	0.009137351
Bo2g006810	Bo9g177250	-0.8904404	0.001285653	4CL3	MSTRG.29785	-0.8004946	0.009549796
Bo8g081770	Bo9g172240	-0.8902813	0.001291991	Bo6g120830	Bo8g081770	-0.8004904	0.009550453
4CL3	MSTRG.15380	-0.8896182	0.001318642	Bo6g117860	Bo9g177250	-0.8003333	0.009575228
Bo8g081770	IQD16	-0.888291	0.001373126	4CL3	Bo3g004170	-0.7982357	0.009910151
Bo5g009840	Bo9g177250	-0.8876709	0.001399112	Bo5g131870	Bo9g177250	-0.7958877	0.01029435
Bo6g106910	Bo9g177250	-0.881647	0.00166957	Bo3g184380	Bo8g081770	-0.7944338	0.01053721
Bo4g195410	Bo8g081770	-0.8796555	0.001766405	Bo3g034260	Bo9g177250	-0.7914662	0.01104485
Bo8g081770	RIP2	-0.8763767	0.001934204	4CL3	Bo7g118740	-0.7893449	0.01141765
Bo2g006660	Bo8g081770	-0.8746323	0.002027824	Bo6g032890	Bo8g081770	-0.789135	0.01145498
Bo8g081770	SKS1	-0.8737642	0.002075562	4CL3	Bo5g009840	-0.7885209	0.01156471
Bo3g017510	Bo9g177250	-0.87131	0.002214716	4CL3	Bo3g017510	-0.788395	0.01158728
Bo7g118740	Bo9g177250	-0.8711408	0.002224541	4CL3	Bo9g181830	-0.7802225	0.01311732
Bo3g163990	Bo8g081770	-0.8686646	0.00237179	4CL3	PFK3	-0.7776816	0.01361925
Bo8g081770	Bo9g153840	-0.8669514	0.002477518	Bo6g117860	Bo8g081770	-0.7762385	0.01390997

Bo3g004170	Bo9g177250	-0.8665979	0.002499732	4CL3	DIN11	-0.7760634	0.01394554
Bo9g177250	DIN11	-0.863608	0.002693136	4CL3	Bo6g120830	-0.769442	0.01533511
Bo8g081770	MSTRG.29785	-0.8562137	0.003215307	4CL3	Bo9g169650	-0.7675178	0.0157556
Bo6g106910	Bo8g081770	-0.854325	0.003359062	Bo1g134740	Bo8g081770	-0.7628252	0.01681315
Bo1g039320	Bo8g081770	-0.8543202	0.003359436	Bo1g016660	Bo9g177250	-0.761213	0.01718711
Bo8g081770	MSTRG.36727	-0.8542348	0.003366032	4CL3	Bo7g093130	-0.7608027	0.01728316
Bo7g093130	Bo9g177250	-0.852708	0.003485597	Bo3g153060	Bo8g081770	-0.7496205	0.02004016
Bo6g072960	Bo8g081770	-0.8521604	0.003529176	4CL3	Bo6g032890	-0.7437223	0.02160513
Bo3g021660	Bo8g081770	-0.852152	0.003529855	4CL3	Bo3g184380	-0.7415393	0.02220422
Bo2g006810	Bo8g081770	-0.8502741	0.003682172	Bo5g131870	Bo8g081770	-0.7367441	0.02355849
Bo9g177250	Bo9g181830	-0.8495976	0.003738133	4CL3	Bo1g134740	-0.7359974	0.02377412
4CL3	MSTRG.6090	-0.8488836	0.003797827	Bo3g034260	Bo8g081770	-0.7344615	0.02422181
Bo9g169650	Bo9g177250	-0.8480801	0.003865781	4CL3	Bo3g153060	-0.7231776	0.02768175
Bo6g120830	Bo9g177250	-0.8478605	0.003884496	4CL3	Bo5g131870	-0.7026272	0.03478437
4CL3	MSTRG.5945	-0.8449058	0.004142404	4CL3	Bo6g117860	-0.7016765	0.03513883
4CL3	ENODL14	-0.8446165	0.004168272	Bo1g016660	Bo8g081770	-0.7006586	0.03552092
Bo9g177250	PFK3	-0.8441267	0.004212315	4CL3	Bo3g034260	-0.690992	0.03928443
4CL3	Bo9g172240	-0.841599	0.004444698	4CL3	Bo1g016660	-0.6870951	0.04087155
